# A knowledge‐based approach to automated planning for hepatocellular carcinoma

**DOI:** 10.1002/acm2.12219

**Published:** 2017-11-15

**Authors:** Yujie Zhang, Tingting Li, Han Xiao, Weixing Ji, Ming Guo, Zhaochong Zeng, Jianying Zhang

**Affiliations:** ^1^ Department of Radiation Oncology Zhongshan Hospital Fudan University Shanghai China; ^2^ Department of Radiation Oncology EYE& ENT Hospital Fudan University Shanghai China

**Keywords:** Auto‐Planning, knowledge‐based, liver cancer

## Abstract

**Purpose:**

To build a knowledge‐based model of liver cancer for Auto‐Planning, a function in Pinnacle, which is used as an automated inverse intensity modulated radiation therapy (IMRT) planning system.

**Methods and Materials:**

Fifty Tomotherapy patients were enrolled to extract the dose–volume histograms (DVHs) information and construct the protocol for Auto‐Planning model. Twenty more patients were chosen additionally to test the model. Manual planning and automatic planning were performed blindly for all twenty test patients with the same machine and treatment planning system. The dose distributions of target and organs at risks (OARs), along with the working time for planning, were evaluated.

**Results:**

Statistically significant results showed that automated plans performed better in target conformity index (CI) while mean target dose was 0.5 Gy higher than manual plans. The differences between target homogeneity indexes (HI) of the two methods were not statistically significant. Additionally, the doses of normal liver, left kidney, and small bowel were significantly reduced with automated plan. Particularly, mean dose and V15 of normal liver were 1.4 Gy and 40.5 cc lower with automated plans respectively. Mean doses of left kidney and small bowel were reduced with automated plans by 1.2 Gy and 2.1 Gy respectively. In contrast, working time was also significantly reduced with automated planning.

**Conclusions:**

Auto‐Planning shows availability and effectiveness in our knowledge‐based model for liver cancer.

## 1 INTRODUCTION

In recent decades, as a standard technique for external beam radiotherapy treatment (RT), IMRT has been widely considered as a safe and effective approach to the cure of hepatocellular carcinoma.^1^, ^2^, ^3^, ^4^ High quality radiotherapy treatment plan is essential to deliver a sufficiently high dose to the target while sparing healthy tissue. Unfortunately, obtaining high quality plans is a demanding task. There are no standard evaluating rules for a “good” plan, and the plan quality may extremely vary both between planners and treatment centers.^5^, ^6^, ^7^ On the other hand, optimizing an IMRT plan is also a time‐consuming task. Several hours even days per case may be needed to achieve a clinically acceptable plan.^8^, ^9^ In addition, the complex of planning grows rapidly as the number of OARs increases.

One solution for those problems is (semi) automated planning. Several in‐house developed knowledge‐based planning solutions were proposed in the last decade.^10^, ^11^, ^12^, ^13^, ^14^, ^15^, ^16^ Some works assembled with Eclipse RapidPlan were also estimated.^17^, ^18^, ^19^ In contrast with these knowledge‐based approaches, an inverse optimizer module of Pinnacle^3^ version 9.10 called Auto‐Planning was introduced recently.

Several works have been accomplished to testify the effectiveness of Auto‐Planning.^20^, ^21^, ^22^ Its plan quality is comparable with knowledge‐based planning approach employing overlap‐volume histogram.^23^ Auto‐Planning was regarded as a useful tool to perform treatment plans automatically.^20^, ^21^, ^22^, ^23^, ^24^, ^25^ However, its availability for liver cancer is still unknown. This study is the first try to build an Auto‐Planning model for liver cancer.

In previous works,^20^, ^21^ protocol‐specific constraints were proposed. Every constraint of setup protocol usually had a certain value for all test patients. Unlike the cases of previous studies, in which the target located in head and neck region, the dose distribution of liver and other organs are strongly related to tumor size in liver cancer. Differences in constraints between different patients should be considered in a reasonable model. Compared to the models in previous works,^20^, ^21^ the major improvement of our model is its knowledge‐based feature. Geometric‐specific constraints were proposed by extracting the dose–volume histograms (DVHs) information of fifty Tomotherapy patients.

In this work, we tested the probability of performing IMRT treatment plans in Pinnacle^3^ version 9.10 module Auto‐Planning along with our model for liver cancer. A knowledge‐based process with both knowledge database and test database was involved. A unique Auto‐Planning model was produced by extracting dose–volume histograms information of fifty historical Tomotherapy patients, which followed by applying the model to twenty more liver cancer patients for testing.

## 2 METHODS AND MATERIALS

### 2.A Patients’ database

Two databases were used in our study: knowledge database and test database. In both databases, patients with diagnosis of liver cancer, in which targets were highly variable in size and localization in liver, were selected retrospectively and randomly. They were referred to curative radiotherapy for liver tumor without retroperitoneal lymph nodes from 2011 up to now in our institution. The radiotherapy was performed by Siemens Oncor or Tomotherapy.

For knowledge database, fifty Tomotherapy patients were enrolled to extract the DVHs information and to construct the protocol of Auto‐Planning model. Twenty more patients, which were not limited to be clinically treated by Tomotherapy, were chosen additionally to set the test database. Each patient was treated with one dose level, and the prescription dose was normalized to 50 Gy.

### 2.B Manual planning

In order to explore the probability of automated planning for liver tumors, manual IMRT planning (shorted by MA) and automated IMRT planning (shorted by AU) were performed blindly for all twenty test patients in our study. Same machine and treatment planning system, which were Varian Trilogy (10 MV) and Pinnacle^3^ version 9.10 (Philips Radiation Oncology Systems, Fitchburg, WI), were used.

Standard clinical practices were executed to create manual plans by three highly experienced physicists. Machine energy, number of beams, and the angle of each beam were determined according to the best judgments of physicists. Following settings were fixed to ensure that the manual plans were comparable with automatic plans: Optimization type was settled to DMPO; the minimum segment area and the minimum segment MUs were 5 cm^2^ and 5, which were common in an ordinary optimization. The maximum number of segments was not a certain number for every patient. However, it was the same in MA and AU plan for each patient. The needed number of segments for one case was strongly related to the complexity of its treatment plan. One description of this complexity is the ratio between planning tumor volume (PTV) and liver volume (shorted by RTL). We assumed that the needed maximum number of segments is linearly bound to RTL as shown by the formula y = 40 + 50x, in which x stands for RTL and y stands for the maximum number of segments.

### 2.C Automated planning

In automated IMRT planning, an inverse optimizer module of Pinnacle^3^ version 9.10 called Auto‐Planning was involved. Equally divided seven beams started at 0 degree were set in AU plans. Machine settings adopted in AU plans were the same with MA plans, including optimization type, the minimum segment area, the minimum segment MUs and the maximum number of segments.

A universal model was needed to present treatment plans automatically. In previous studies, models were usually based on training processes,^19^, ^20^, ^21^, ^22^ in which only a handful of cases were applied. On the contrary, in our study, the model was created by knowledge‐based method through fifty Tomotherapy treatment plans. In particular, the DVHs information of these fifty plans was extracted to construct the constraints of OARs, including relative volume of liver‐GTV at 5 Gy, 10 Gy, 20 Gy, 30 Gy (V5, V10, V20, V30), mean dose of kidneys and heart, maximum dose of spinal cord planning volume (PRV) 5 mm and esophagus, and the relative volume of stomach and small bowel at 20 Gy (V20) and 15 Gy (V15) respectively.

### 2.D Protocols for Auto‐Planning model

As mentioned in the introduction part, for liver cancer, differences in constraints between different patients should be considered in a reasonable model. In this study, we assumed that the dose features of liver, kidneys and spinal cord were approximately linearly related to RTLs. It was confirmed by the statistics of fifty TomoTharapy patients’ DVHs in the knowledge database. Linear fittings were performed for normal liver (liver‐GTV, in which GTV stands for gross tumor volume), kidneys and spinal cord PRV 5 mm. For normal liver, the intercept of the fitting line was decreased, as well as the fitting line moved lower parallel, to insure that the fitting parameters covered statistical 95% cases of knowledge database. As results, the relationships between relative volume percentages and RTLs for V5, V10, V20, V30 of liver‐GTV were y = 43.19 + 29.49x, y = 30.62 + 27.26x, y = 8.22 + 38.81x and y = 3.15 + 41.52x respectively, in which x stood for RTL and y stood for the relative volume percentage. For kidneys and spinal cord PRV 5mm, the formulas of the fitting lines were y = 211 + 462x, y = 380 + 1465x and y = 1990 + 2005x, respectively, in which x stood for RTL and y stood for mean dose or maximum dose (unit: cGy). However, the dose features of stomach, small bowel, heart and esophagus seemed quite randomly as a function of RTL. It was potentially caused by the varied target localization in liver tumor. As these OARs were less considered in a standard manual plan of liver tumor, we took the medium value of the population as the constraint for each OAR.

The OARs were prioritized by their weights in an ordinary manual optimization. Liver‐GTV and kidneys had high priority and the priorities of other structures were low. Option “Compromise” was set to be “yes” for all OARs. Unlike the dose or volume optimization constraints, which were specific by each patient, the “Priority” and “Compromise” were the same for every patient. The general settings of Auto‐Planning were listed below: tuning balance = 7%, dose fall‐off margin = 2.6 cm, hot‐spot maximum goal = 106%, and using cold‐spot ROIs.

All twenty test patients were simulated using this uniform protocol and parameters with no exception. Here we present an example for better understanding of our model. For instance, the RTL of one patient in the test database was 0.55. The optimization constraint of this patient was shown in Table 1.

**Table 1:** The protocol details of Auto‐Planning for one patient. The “Priority” and “Compromise” were the same for every patient. However, the “Constraints” were specific by different patients

Organs at risk	Constraint	Priority	Compromise
Liver‐GTV	V5 < 59%	High	Yes
Liver‐GTV	V10 < 46%	High	Yes
Liver‐GTV	V20 < 30%	High	Yes
Liver‐GTV	V30 < 26%	High	Yes
Left kidney	Mean dose < 464 cGy	High	Yes
Right kidney	Mean dose < 1184 cGy	High	Yes
Spinal cord PRV 5mm	Maximum dose < 3090 cGy	Low	Yes
Stomach	V20 < 15%	Low	Yes
Small bowel	V15 < 35%	Low	Yes
Heart	Mean dose < 630 cGy	Low	Yes
Esophagus	Maximum dose < 3863 cGy	Low	Yes

### 2.E Plan comparison and statistics

To compare MA and AU plans for every patient in the test database, the following structures were considered and analyzed: PTV, liver‐GTV, kidneys, spinal cord, stomach, small bowel, heart, and esophagus. Mean doses and DVHs of PTV and OARs were calculated for both MA and AU plans. The maximum dose of spinal cord and the volume of liver‐GTV at 15 Gy (V15) were also taken into consideration. To evaluate target dose distributions, target conformity index (CI=(V_prescription in PTV_/V_PTV_)*(V_prescription in PTV_/V_prescription_)) and homogeneity index (HI = D_2%_/D_98%_) were calculated as well.^26^

Mean doses of all structures, maximum dose of spinal cord, V15 of liver‐GTV, as well as CI and HI of PTV were tested using paired *t* test for statistical analysis. The cutoff of *P*‐value accepted as significant was <0.05. In addition, the average DVHs for all OARs and three dose distribution samples were performed as well.

Furthermore, a “double‐blind test” was performed as a supplement of statistical analysis. All AU and MA plans were blind‐reviewed by three physicians to determine which plan was better for each patient in clinical opinion. The evaluations were also blind from one physician to another.

## 3 RESULTS

### 3.A Target features

In this work, the target features were described by CI, HI and mean dose of the PTV. As the statistical data shown in Table 2, the average target CIs of MA and AU plans were 0.84 ± 0.07 and 0.87 ± 0.03 respectively. The AU plans performed better in target CI, as their average CI was closer to the standard value 1 (*P* < 0.05). On the contrary, MA plans performed better than AU plans in target HI, and the values for them were 1.07 ± 0.03 and 1.09 ± 0.02 respectively. However, the difference of HI was not statistically significant. It could be considered that MA and AU plans performed no difference in HI. In contrast, the mean target dose of AU plans, which was 51.8 Gy, was slightly higher than the mean target does of MA plans, which was 51.3 Gy (*P* < 0.05).

**Table 2:** Paired *t*‐test analysis of PTV and OARs. The statistically significant *t*‐test numbers and related mean values were bolded

	MA plan		AU plan		*t*‐test
	Mean	StDev	Mean	StDev	
CI of PTV	**0.84**	0.07	**0.87**	0.03	**0.033**
HI of PTV	1.07	0.03	1.09	0.02	0.093
Mean dose of PTV (cGy)	**5132.1**	50.7	**5182.1**	34.8	**0.001**
Mean dose of liver‐GTV (cGy)	**1767.1**	640.9	**1630.2**	572.6	**<0.001**
V15 of liver‐GTV (cc)	**487.2**	199.0	**441.3**	163.7	**0.004**
Mean dose of left kidney (cGy)	**308.1**	316.4	**191.2**	155.8	**0.020**
Mean dose of right kidney (cGy)	820.7	706.9	722.2	661.6	0.071
Mean dose of spinal cord (cGy)	1233.3	613.6	1085.9	660.3	0.129
Maximum dose of spinal cord (cGy)	2739.8	963.2	2460.9	1212.4	0.172
Mean dose of stomach (cGy)	691.3	438.5	675.5	492.1	0.792
Mean dose of small bowel (cGy)	**885.8**	716.4	**676.8**	627.3	**0.012**
Mean dose of heart (cGy)	427.4	679.3	403.9	677.9	0.339
Mean dose of esophagus (cGy)	943.0	879.0	850.9	881.1	0.348

### 3.B Organs at risk

**Figure 1:**
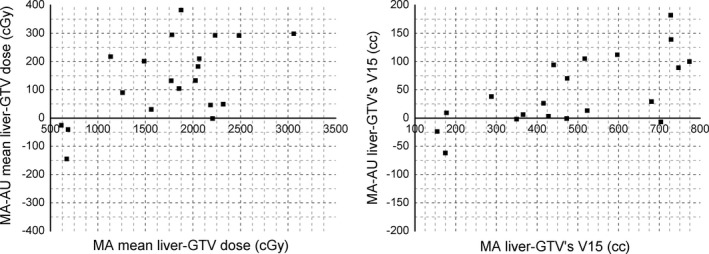
Mean liver‐GTV dose and liver‐GTV V15 differences as a function of the MA mean liver‐GTV dose and liver‐GTV V15. Positive values indicated reduced mean dose and liver‐GTV V15 in AU plans, while negative values indicated reduced mean dose and liver‐GTV V15 in MA plans.

The OAR sparing was also compared between MA and AU plans. Mean doses of all OARs, liver‐GTV V15, and maximum spinal cord dose were summarized in Table 2. For liver‐GTV, left kidney and small bowel, their mean doses were significantly reduced in AU plans (Table 2). In particularly, mean dose and V15 of liver‐GTV were 1.4 Gy and 40.5 cc lower in AU plans, respectively, compared to those in MA plans (*P* < 0.05). Comparisons of mean liver‐GTV doses and liver‐GTV V15s for twenty test patients were illustrated in Fig. 1. Auto‐Planning was able to reduce mean doses and V15s of liver‐GTV in 16 cases (80%) and 15 cases (75%) respectively. In contrast, mean doses of left kidney and small bowel were reduced with AU plans by 1.2 Gy and 2.1 Gy respectively (*P* < 0.05). All Auto‐Planning cases with higher mean doses or V15s of liver‐GTV were clinical acceptable.

The mean dose and maximum dose of spinal cord was reduced with AU plans by 1.5 Gy and 2.8 Gy respectively. The mean doses of right kidney, stomach, heart, and esophagus were reduced in AU plans by 1.0 Gy, 0.2 Gy, 0.2 Gy, and 0.9 Gy respectively. However, as shown in Table 2, statistical data for these OARs were not significant.

**Figure 2:**
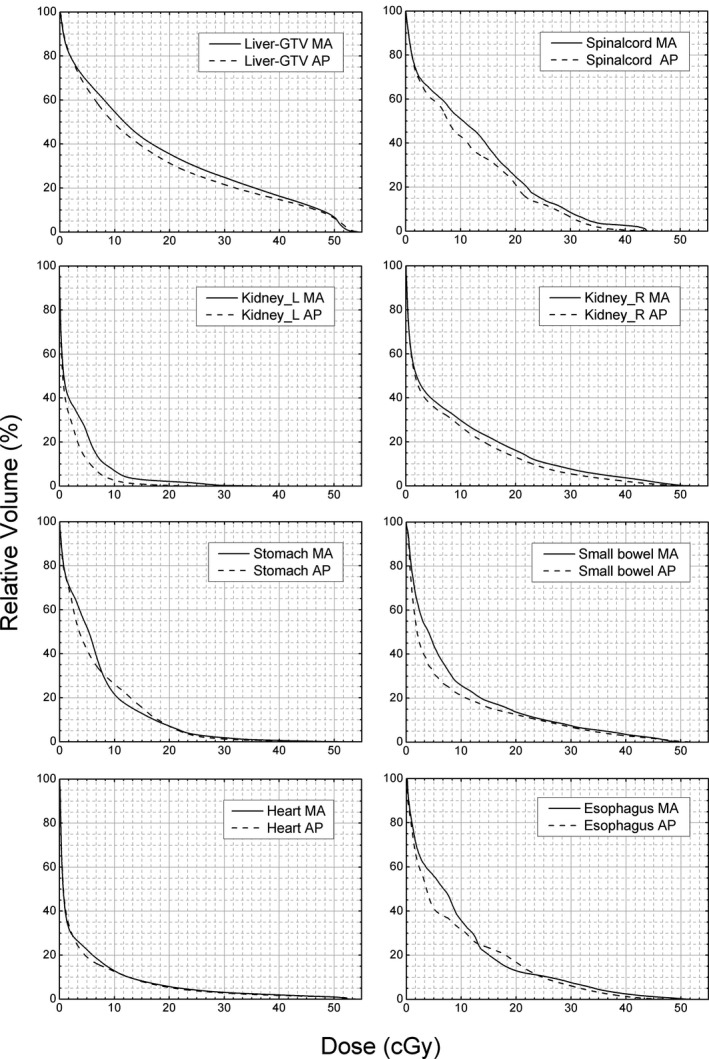
Mean DVH of eight OARs for all 20 patients in test database. The solid lines stand for MA plans and the dashed lines stand for AU plans.

For further analysis, mean DVHs of all OARs were illustrated in Fig. 2. The solid lines stand for MA plans, and the dashed lines stand for AU plans. For liver‐GTV, spinal cord, both kidneys, small bowel, and heart, reduced mean DVHs with AU plans covered almost the whole dose range (0~55 Gy). In contrast, the mean DVHs of stomach and esophagus twisted between solid and dashed lines. The mean relative volume was lower in MA plan than in AU plan from 8 Gy to 20 Gy for stomach and 13 Gy to 24 Gy for esophagus. For the rest region of dose range, the mean relative volume of AU plan was lower than MA plan.

### 3.C Dose distribution samples

**Figure 3:**
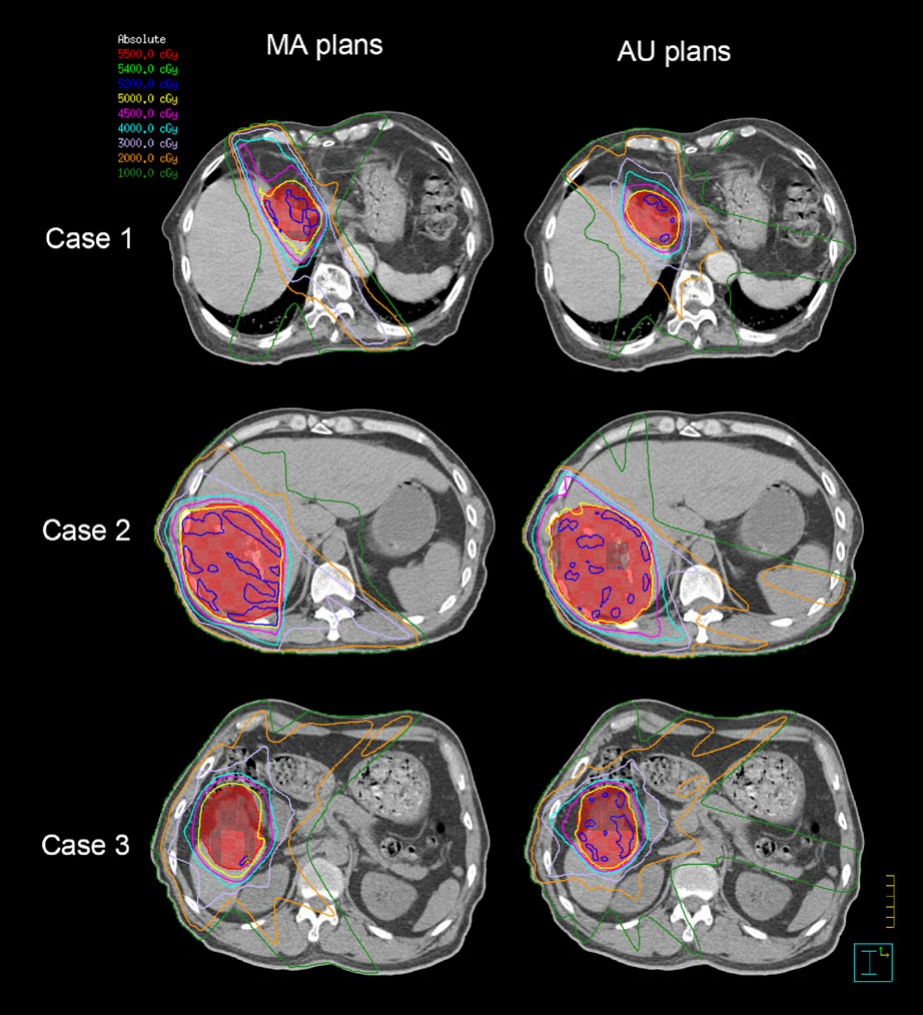
Dose contours comparison of three patients in transverse. Iso‐dose curves are 5500 cGy (red), 5400 cGy (green), 5200 cGy (blue), 5000 cGy (yellow), 4500 cGy (purple), 4000 cGy (skyblue), 3000 cGy (lavender), 2000 cGy (orange), 1000 cGy (forest). The PTV is shown in red with both contour and colorwash.

Three cases in transverse were selected to estimate dose distribution (Fig. 3). Multidose contours were shown in different colors. In general, axial isodose distributed smoother in AU plans. The prescription dose (50 Gy, yellow) was kept closer to the PTV in AU plans for these three patients. It was confirmed by better CIs, which were reduced with AU plans by 0.17, 0.03, and 0.15, respectively, of these three cases.

### 3.D Double‐blind test by physicians

**Figure 4:**
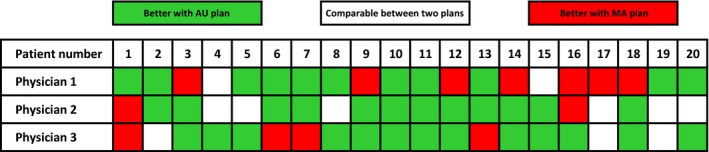
Double‐blind test by three physicians. Each column represents the results for a patient. Each row represents the results for one physician. In green, AU plans were chosen by physicians. In red, MA plans were chosen by physicians. In white, AU and MA plans were considered to be comparable by physicians.

In addition to statistical analysis, a “double‐blind test” was performed to determine whether AU or MA plan was better for each patient in clinical opinion. All AU and MA plans were examined by three physicians. The results were shown in Fig. 4. Each column represents the results for a patient, and each row represents the results for one physician. The patients whose AU plans were better were marked by green. The patients whose MA plans were better were marked by red. The patients whose AU and MA plans were comparable were marked by white.

For the first physician, 55% of the twenty AU plans were chosen as the better plan, 35% of the twenty MA plans were chosen as the better plan, and the rest 10% of AU and MA plans were considered to be comparable. For the second physician, 60% of the twenty AU plans were chosen as the better plan, 10% of the twenty MA plans were chosen as the better plan, and the rest 30% of AU and MA plans were considered to be comparable. For the third physician, 65% of the twenty AU plans were chosen as the better plan, 20% of the twenty MA plans were chosen as the better plan, and the rest 15% of AU and MA plans were considered to be comparable. The results were consistent with the statistical comparison.

## 4 DISCUSSION

### 4.A Target size and localization

Unlike the cases of previous studies,^14^, ^15^, ^18^, ^19^, ^20^, ^21^ in which the targets were located in head and neck region or pelvic cavity, for liver cancer, tumors were highly variable in target size and localization. In our model, we considered the relationship between dose distributions of OARs and target size by introducing the ratio between PTV and liver volume. Positive results indicated their strong correlation.

However, the effect of varied target localization was not taken into account in our model. A simple analysis was performed by dividing 20 test patients into two groups according to their target localization. The lateral distance between centers of PTV and liver was defined as the index to differentiate target localization. Positive and negative values indicated localized deviation to left and right.

Unfortunately, only four patients were divided into left target group while other sixteen patients’ targets deviated to right. Paired *t*‐tests were performed for each group, and only the results for right target group were statistically significant. For left target group, the CI, HI and mean dose of PTV were 0.89, 1.08, 5173 cGy with AU plans and 0.82, 1.09, 5152 cGy with MA plans. The mean dose and V15 of liver‐GTV were reduced by 1.0 Gy and 23.0 cc with AU plans. For right target group, the CI, HI and mean dose of PTV were 0.87, 1.09, 5184 cGy with AU plans and 0.84, 1.07, 5127 cGy with MA plans. The mean dose and V15 of liver‐GTV were reduced by 1.5 Gy and 51.6 cc with AU plans (*P* < 0.05, except CI). The differences between target localizations were not significant, albeit, further identification would be needed for this assumption.

### 4.B Further analysis of normal liver

In liver cancer, liver‐GTV is the most important organ at risk. Fogliata and her collogues’ work,^17^ in which closed‐loop and open‐loop validations of advanced hepatocellular cancer were experienced by Eclipse RapidPlan, showed qualitative and quantitative equivalence of normal liver between the clinical and the test plans. Our work shed new light on liver cancer treatment. As indicated in section results, both mean dose and V15 of liver‐GTV were reduced with AU plans.

Detailed mean dose and V15 comparison between MA and AU plans for twenty test patients were illustrated in Fig. 1. Similar results were observed in these two most critical evaluation conditions of liver‐GTV. For liver‐GTV mean dose, only four patients (20%) had lower value with MA plans. The largest reduction in liver‐GTV mean dose with AU plan compared to MA plan was 3.8 Gy (23%). For liver‐GTV V15, only five patients (25%) had lower value with MA plans. The largest reduction in liver‐GTV V15 with AU plan compared to MA plan was 182 cc (29%). Similar results for parotid, swallowing muscles and oral mucosa were observed by Krayenbuehl et al in their work of head and neck cancer by Auto‐Planning.^20^

### 4.C Comparisons of the best and worst scenarios

**Figure 5:**
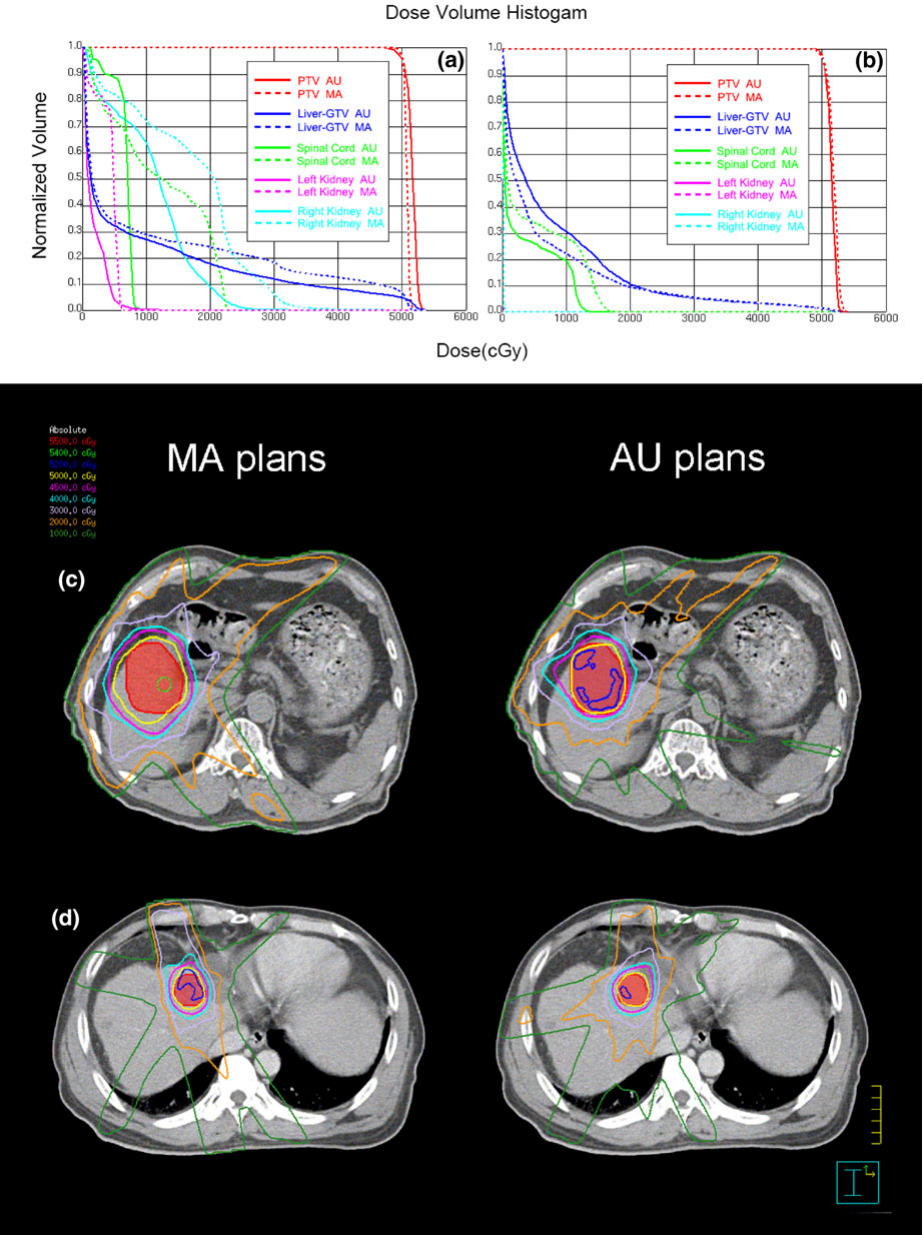
The DVHs and dose distributions of the best scenario (a, c) and worst scenario (b, d) of Auto‐Planning. In (a) and (b), the solid lines stand for AU plans and the dashed lines stand for MA plans. Different colors stand for PTV(red) and different OARs. The OARs shown here were liver‐GTV(blue), spinal cord(green), left kidney(purple) and right kidney(skyblue). In (c) and (d), Iso‐dose curves are 5500 cGy (red), 5400 cGy (green), 5200 cGy (blue), 5000 cGy (yellow), 4500 cGy (purple), 4000 cGy (skyblue), 3000 cGy (lavender), 2000 cGy (orange), 1000 cGy (forest). The PTV is shown in red with both contour and colorwash.

For further analysis, we also presented the DVHs for the best and worst scenarios of Auto‐Planning. As there are no standard criteria for a “good” plan, these two plans were chosen by our best judgment. The DVHs of the best and worst scenarios were shown in Figs. 5(a) and 5(b) respectively. The solid lines stand for AU plans and the dashed lines stand for MA plans. Different colors stand for PTV(red) and different OARs. The OARs shown in Fig. 5 were liver‐GTV(blue), spinal cord(green), left kidney(purple) and right kidney(skyblue). The differences between other OARs were not significant.

In the best case, the target CIs of MA and AU plans were 0.74 and 0.89, and the target HIs of MA and AU plans were 1.04 and 1.07. The AU plan performed much better in CI, while the MA plan performed better in HI. However, the maximum dose of PTV in AU plan was clinically acceptable, as it was lower than 110% of the prescription dose. In addition, the doses of liver‐GTV, spinal cord, and both kidneys in AU plan were all significantly lower than the doses in MA plan.

In the worst case, the target CIs of MA and AU plans were 0.76 and 0.81, and the target HIs of MA and AU plans were 1.07 and 1.06. The AU plan performed slightly better both in CI and HI. The maximum dose of heart was also lower in AU plan. However, the mean dose and V15 of liver‐GTV were significantly higher in AU plan.

The dose distributions of the best and worst scenarios were illustrated in Figs. 5(c) and Fig. 5(d). Multi dose contours were shown in different colors. The prescription dose (50Gy, yellow) was kept closer to the PTV in AU plans for both patients. However, larger volume irradiated by low dose was revealed both in Figs. 5(b) and Fig. 5(d) with AU plan.

Our analysis shows that in the best case, AU plan was much better than MA plan, whereas in the worst case, the difference between AU and MA plans were not remarkable. The AU plan was still clinically acceptable even in the worst case. These conclusions were confirmed by physicians.

### 4.D Constraints for OARs in Auto‐Planning model

Spinal cord was usually regarded to have higher priority than target and a noncompromise constrain could be set.^20^, ^21^ However, in our Auto‐Planning model, option “compromise” was set to be “yes” for all constrains without any exception. As the cutoff of constrain for maximum spinal cord dose was not the certain value of tolerance dose in our protocol, it would be unreasonable to set as a noncompromise constrain.

In the other hand, for liver cancer, spinal cord was usually not very close to the target and the maximum spinal cord dose was easy to keep below tolerance dose. In our study, for every test patient, AU plan was able to keep the maximum spinal cord dose below tolerance, which was less than 1 cc related the dose above 45 Gy. An additionally optimization goal of maximum spinal cord dose with noncompromise maybe more secure. But in Auto‐planning, different compromise settings of constrains for one OAR is not allowed. However, minor manual fine‐tuning of plan could be performed in optimized module. In Hansen and his collogues’ work, minor manual modifications were performed for every patient to achieve better AU plans.^21^

The maximum stomach and small bowel doses were also less considered in our model. As the prescription dose was normalized to 50 Gy for all patients in our study, the tolerance dose for maximum stomach and small bowel doses were easy to reach. However, in clinical cases, the prescription dose may be 54 Gy or 60 Gy, and above. In that case, additional manual modification would be needed to constrain the maximum dose of stomach and small bowel.

### 4.E MU comparison and planning time

The MUs of AU and MA plans for each patient were checked. For 80% of the patients, the MUs of AU plans were higher than MA plans. Comparing with the MA plans, the ratio of average MU increase with AU plans was 10.59%. The result was generally consistent with the result in Hansen and his collogues’ work,^21^ in which the ratio of average MU increase with AU plans was about 20.83%. The MU increase with AU plans was considered as an acceptable cost to improving plan quality.

The approximate working time required for optimizing one MA or AU plan was evaluated. As in a manual planning process, accessorial rings and other helped structures may be needed, the time to construct these counters was also included in the MA working time. Hours up to days of an experienced physicist or dosimetrist were needed for performing one MA plan. Several optimization cycles were needed in this process. On the contrary, the time needed for one AU plan was only about one hour with only one optimization cycle. And as the protocol was settled without any changeable parameter, it could be executed by a junior.

In Krayenbuehl and his collogues’ work,^20^ the average effective working time was 3.8 ± 1.1 min with AU plans in comparison to 48.5 ± 6.0 min with MA plans. In contrast, average operator time of 32 and 64 min with AU and MA plans were evaluated by Hansen et al.^21^ Compared to these two works of Auto‐Plannning, the planning time in our study was obviously much longer for both MA and AU plans, probably because that 0.2 and CC convolution were used as the dose grid resolution and the dose computation algorithm in this work. Further attempts showed that planning time could be reduced to approximately 10 min per AU plan by using 0.4 as dose grid resolution and adaptive convolve as dose computation algorithm.

### 4.F Is the model universal effective?

Compared to the models in previous works,^20^, ^21^ the major improvement of our model is its knowledge‐based feature. Instead of training process, which is usually applied limited in the same planning system and machine, a knowledge‐based method is applied in our study. Fifty Tomotherapy patients were enrolled to extract the DVHs information and construct the geometric‐specific protocol for Auto‐Planning model.

Tomotherapy was widely accepted as an advanced approach for performing IMRT. It is reasonable to regard its results as guides for linear accelerator IMRT. Furthermore, as different planning systems and machines were involved, we assumed that our model was independent from the treatment planning systems and linear accelerator machines. More works would be performed to testify this assumption.

### 4.G Limitation

In our work, geometric‐specific constraints for Auto‐Planning engine were proposed. Our study is the first try to build an Auto‐Planning model combining with knowledge‐based approach. The results were promising. However, there were still some limitations. First, in our study, the index used to indicate geometric feature was the RTL (volume ratio). It worked in liver cancer, but this index may be too simplified on other disease sites. More complicated indexes, such as the OVH and DTH which proposed in previous works,^10^, ^11^, ^12^, ^13^, ^15^, ^16^ may improve the overall performance of AU plan.

Another limitation of our model is that the knowledge database we used may not be large enough. The purpose of this work is to test the possibility of employing geometric‐specific constraints in Auto‐planning. The extracted data of fifty Tomotherapy patients worked fine in this study. However, for about 25% cases in the test database, MA plans were better than AU plans. Increasing number of patients in the knowledge database may further improve the quality of AU plans.

Additionally, as different planning systems and machines were involved, we assume that our model is independent from the treatment planning systems and linear accelerator machines. This is the advantage of our model. However, it may also cause problems. The follow‐up study is still in process.

## 5 CONCLUSION

In conclusion, Auto‐Planning is available and effective with our novel knowledge‐based model for hepatocellular carcinoma. Seventy patients with diagnosis of liver cancer referred to curative radiotherapy for liver tumor without retroperitoneal lymph nodes were involved. Statistically significant results showed that automated plans performed better in target CI, while mean target dose was 0.5 Gy higher with automated plans. The differences in target HI were not statistically significant. Additionally, the doses of liver‐GTV, left kidney and small bowel were significantly reduced with automated plan. Reduced mean DVHs with AU plans covered most OARs. In contrast, working time was also significantly reduced with automated planning.

## ACKNOWLEDGMENTS

This research was technically supported by Philips Radiation Oncology Systems Pinnacle3 version 9.10.

## CONFLICT OF INTEREST

The authors declare no conflict of interest.
